# Complexities of JC Polyomavirus Receptor-Dependent and -Independent Mechanisms of Infection

**DOI:** 10.3390/v14061130

**Published:** 2022-05-24

**Authors:** Jenna Morris-Love, Walter J. Atwood

**Affiliations:** 1 Department of Molecular Biology, Cell Biology, and Biochemistry, Brown University, Providence, RI 02912, USA; jenna_morris-love@brown.edu; 2 Pathobiology Graduate Program, Brown University, Providence, RI 02912, USA

**Keywords:** polyomaviruses, virus receptors, extracellular vesicles, biogenesis

## Abstract

JC polyomavirus (JCPyV) is a small non-enveloped virus that establishes lifelong, persistent infection in most of the adult population. Immune-competent patients are generally asymptomatic, but immune-compromised and immune-suppressed patients are at risk for the neurodegenerative disease progressive multifocal leukoencephalopathy (PML). Studies with purified JCPyV found it undergoes receptor-dependent infectious entry requiring both lactoseries tetrasaccharide C (LSTc) attachment and 5-hydroxytryptamine type 2 entry receptors. Subsequent work discovered the major targets of JCPyV infection in the central nervous system (oligodendrocytes and astrocytes) do not express the required attachment receptor at detectable levels, virus could not bind these cells in tissue sections, and viral quasi-species harboring recurrent mutations in the binding pocket for attachment. While several research groups found evidence JCPyV can use novel receptors for infection, it was also discovered that extracellular vesicles (EVs) can mediate receptor independent JCPyV infection. Recent work also found JCPyV associated EVs include both exosomes and secretory autophagosomes. EVs effectively present a means of immune evasion and increased tissue tropism that complicates viral studies and anti-viral therapeutics. This review focuses on JCPyV infection mechanisms and EV associated and outlines key areas of study necessary to understand the interplay between virus and extracellular vesicles.

## 1. Introduction

Polyomaviruses were initially considered members of the *Papovaviridae* family, combining papillomaviruses and polyomaviruses together for their clear similarities in genome features and capsid morphology, but around 2000 the two viral families were split to *Papillomaviridae* and *Polyomaviridae* to clarify distinct families [1,2]. The *Polyomaviridae* family currently includes 117 viral species that infect an array of animal species. Of the identified species, 14 are human polyomaviruses with only four associated with disease in humans—BK, JC, Merkel Cell, and *Trichodysplasia spinulosa*-associated polyomaviruses [1]. The first two human polyomaviruses were discovered in 1971—BK and JC polyomaviruses—from patient samples and named after the initials of the respective patients [3,4]. Both BKPyV and JCPyV establish persistent infections in the kidney, but typically only BKPyV is pathologic at this site causing hemorrhagic cystitis and nephropathy [5]. JCPyV is the etiologic agent of the neurodegenerative disease progressive multifocal leukoencephalopathy (PML) and is associated with several other rare neurological diseases [5,6]. Merkel cell polyomavirus (MCPyV) was discovered in 2008 from Merkel Cell carcinoma (MCC) tissue samples and has since been associated with about 80% of MCCs [7,8]. In 2010 *Trichodysplasia spinulosa*-associated polyomavirus (TSPyV) was linked to the eponymous disease after isolation and identification from patient TS spines and lesions [9,10]. Some recent evidence links human polyomaviruses 6 and 7 with pruritic rashes and requires further confirmatory studies [11,12]. Polyomavirus-induced diseases are all associated with immune-compromised and/or immune-suppressed patients, indicating that uncontrolled infections allow for disease progression [13]. While four polyomaviruses are clearly associated with human disease, BKPyV and JCPyV are the best-studied human polyomaviruses and currently the only two identified in association with extracellular vesicles [14,15,16]. JCPyV infection mechanisms and the consequences of EV association will be the focus of this work.

## 2. Progressive Multifocal Leukoencephalopathy

Progressive multifocal leukoencephalopathy (PML) is a rare but rapidly developing, neurodegenerative disease [17,18]. Severely immune-compromised and immune-suppressed patients have the greatest risk for PML development. Though the first cases of PML were associated with lymphoproliferative disorders [19,20], during the HIV/AIDS pandemic PML developed in up to 5% of patients and was considered an AIDS-defining disease [21,22]. Introduction of antiretroviral therapies against HIV has reduced the prevalence of PML and increased survival statistics for patients but PML survivors often suffer debilitating symptoms [20]. In the early 2000s the monoclonal antibody therapy natalizumab (brand name Tysabri) used to treat multiple sclerosis was the first immune-suppressive therapy found to increase PML risk [23,24,25]. Since then, additional immune-suppressive and disease-modifying therapies have been linked with increased risk for PML [26,27]. Patient risk for PML also increases the longer someone is treated with immune-suppressive therapies [28].

PML disease progression is marked by lytic destruction of oligodendrocytes and astrocytes [17,29]. Destruction of the myelin-producing oligodendrocytes accelerates neurodegeneration and presents characteristic asymmetrical lesions. Diagnosis is based on confirmation of such lesions using magnetic resonance imaging and evaluation of JCPyV titer from patient CSF samples [30,31,32]. Symptoms include hemiparesis, ataxia, disrupted motor function, and sensory deficits [6,30,33,34]. There is currently no licensed anti-JCPyV treatment to help PML patients. Treatments are centered around ceasing PML disease progression via immune reconstitution, but this has a risk for immune reconstitution inflammatory syndrome (IRIS). The majority of natalizumab treated patients and ~20% of HIV/AIDS patients are at risk for IRIS [35,36]. PML associated IRIS has ~28% mortality rate [37]. Further research into JCPyV dissemination to and within the central nervous system, and at a subcellular level is needed to help prevent CNS infection and disease progression to better treat at-risk patients.

## 3. JC Polyomavirus Genome Organization

**Figure 1:**
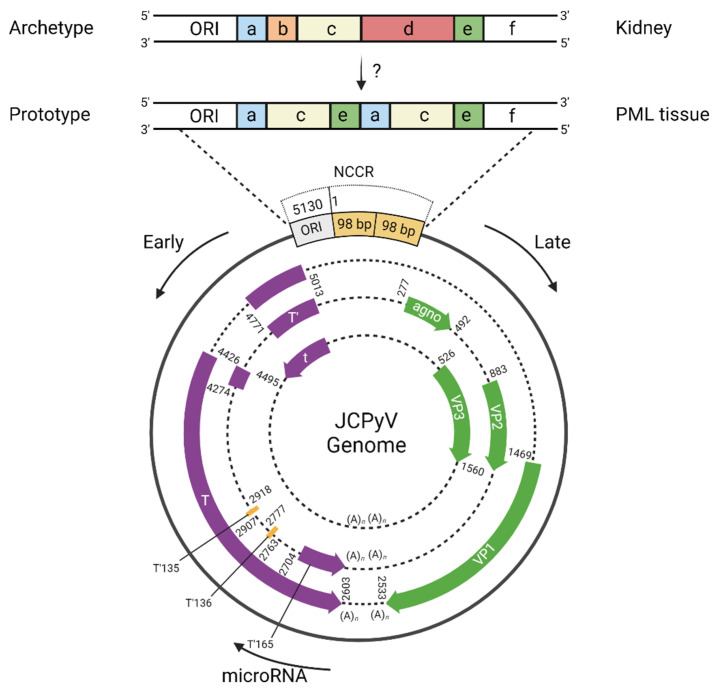
JCPyV genome organization. JCPyV archetype (top sequence, Cy strain) verses prototype, or rearranged, (bottom sequence, Mad-1 strain) non-coding control region (NCCR). NCCRs contain a combination of blocks a-f that contain enhancer elements and a bi-directional promoter. Archetype NCCR is composed of blocks a, b, c, d, e, f whereas prototype Mad-1 NCCR is composed of blocks a, c, e, a, c, e, f. The mechanism and location of rearrangement is not defined. Archetype is mostly found in the kidney while prototype is associated with PML tissue. The genome expresses nine proteins split into early versus late. Early expression (purple genes) includes large T-antigen, small t-antigen, and the minor T’ proteins T’135, T’136, and T’165. Late expression (green genes) includes agnoprotein, VP1, VP2, and VP3. The single viral microRNA is expressed late and has a seed region complementary to T, as depicted. Schematic created with BioRender.com (accessed on 18 April 2022).

Polyomaviruses contain viral minichromosomes that are double-stranded, closed circular DNA genomes wrapped around host-derived histone proteins. The JCPyV genome is approximately 5130 bp with a variable non-coding control region (NCCR) that expresses 9 proteins and 1 microRNA [29,38]. JCPyV NCCRs are classified as archetype or prototype (also referred to as rearranged). JCPyV archetype (Cy strain) NCCR is organized with an origin of replication (ORI) followed by blocks termed A through F that contain enhancer elements and a bi-directional promoter for early and late gene expression (Figure 1) [29,38,39]. Prototype (Mad-1 strain) and prototype-like JCPyV NCCRs are rearranged with some combination of deletion(s) and duplication(s) of blocks A through F that increase the transcription binding sites for both early and late gene expression [40,41]. Mad-1 (named for its discovery in Madison, Wisconsin) contains a deletion of blocks B and D, and duplication of blocks A, C, and E (depicted in Figure 1). JCPyV early gene expression includes the regulatory proteins large T antigen, small t antigen, and three T prime (T’) proteins while late gene expression includes agnoprotein and the structural proteins viral protein 1 (VP1), VP2, and VP3 (Figure 1) [29,42].

Archetype NCCR is classified as the transmissible form of JCPyV since it is detected across healthy patients and contains full genetic information to create prototype and prototype-like viruses detected in PML patients [43,44,45]. Archetype establishes low-level, persistent infections in the kidney and is mostly detected in kidney and urine from healthy and PML patients [46,47]. Archetype can infect and might also persist in bone marrow-derived cells, stromal cells, and brain tissue [48,49,50,51,52,53]. However, archetype is rarely found associated with PML tissue, whereas rearranged JCPyV is neuropathogenic and mostly detected in the cerebral spinal fluid (CSF), brain, and blood [49,51]. Mad-1 is the representative rearranged JCPyV strain and is often studied in laboratory settings.

**Figure 2:**
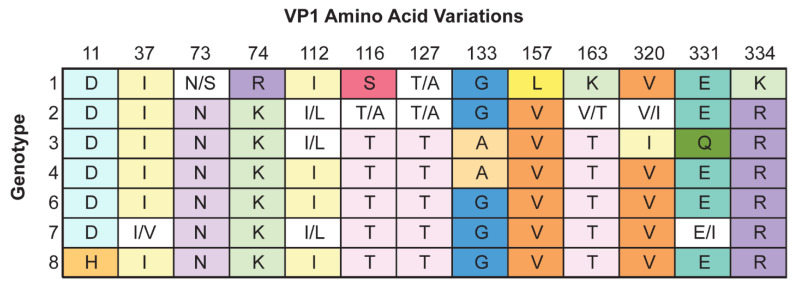
VP1 amino acid variations between genotypes. Genotypes (rows) and key VP1 sequence locations (columns, top) and associated amino acid differences are noted. One color was assigned to each amino acid. White signifies multiple possibilities.

Based on sequence analyses there are 7 genotypes of JCPyV that can be correlated with geographic populations [54,55,56,57,58]. Sequence analyses are often based on the sequence of the major viral capsid protein (VP1), but some studies have adjusted their methods to include whole genome phylogenetic analysis [54,59]. There is no identified correlation between VP1 sequence and neurotropism, but there is a clear association of rearranged NCCRs and neuropathogenic JCPyV [43,44,60,61]. Interestingly, research also shows a propensity for prototype JCPyV variants to develop from JCPyV types 1–4. Most rearranged variants detected in PML patient samples are derived from types 1 and 2, while types 4 and quite rarely type 3 also contribute to PML cases [43,44,54,62,63,64,65,66]. Likelihood of rearrangement is as follows: types 1 > 2 > 4 > 3 [54,59]. Figure 2 outlines major amino acid changes in and around the major VP1 binding pocket of the different genotypes.

## 4. Capsid Morphology and Assembly

Polyomavirus particles are composed of 72 capsomeres assembled into icosahedrons with *T* = *7d* symmetry that measure approximately 40–50 nm in diameter [67,68,69]. One capsomere is constructed of five major viral capsid protein (VP1) molecules that assemble into pentamers with one minor viral capsid protein (VP2 or VP3) attached at the center [70,71,72]. VP1 makes up the entire outer face of a virion making it the major mediator of virus-host interactions. All well-studied human PyVs attach to sialic acid-containing receptors at the cell surface before entry and hemagglutinate red blood cells, though exact receptors differ between viral species and strains [73,74,75].

## 5. JC Polyomavirus Receptor-Dependent Infection

JCPyV utilizes a required two-step mechanism to infect a target cell, (1) attachment followed by (2) entry. Nonenveloped virions require the attachment receptor known as lactoseries tetrasaccharide C (LSTc) for infection [76,77]. Researchers used VP1 pentamers (type 1) in a glycan array and found VP1 strongly binds LSTc. The tight interaction between LSTc and VP1 allowed for crystallization and characterization of the complex. Researchers defined exact amino acid contacts between the VP1 binding pocket and the α-2,6-linked sialic acid and neighboring GlcNac of LSTc. Findings were confirmed using virus binding and infection assays [76]. Interestingly, deep sequencing of viral genomes from PML patients found a host of viral quasi-species containing mutations in this sialic-acid binding pocket of VP1 [62,63,78]. Most viral species were derived from genotypes 1 and 2. The VP1 mutations coincided with critical contact locations previously identified and destroy the sialic-acid binding capacity of these viruses in vitro [76,79]. Confounding this discovery, oligodendrocytes and astrocytes were found to lack the necessary sialic acid-containing attachment receptor LSTc, and JCPyV (genotype 1) was incapable of binding these cell types at detectable levels in patient tissue sections [80].

Alternative attachment factors examined include gangliosides that serve as major attachment factors for other well-studied polyomaviruses like BKPyV and SV40 [77,81,82]. Gangliosides are glycosphingolipids that carry sialic acid receptors and are plentiful in brain tissue [83]. These attributes make gangliosides good candidate receptor species for JCPyV. Studies using JCPyV virus-like particles (VLPs) of the genotype 1, 2, or 3 genetic background found JCPyV can attach to gangliosides [62,81,84]. However, direct comparison between JCPyV types 1 and 3 (using pentamers and live virus) demonstrated while each can bind gangliosides loosely, both genotypes have greater affinity for LSTc [77]. Stroh and colleagues showed that reincubation with ganglioside GM1 inhibited virus (type 1 and 3) infection, but less so than blocking with LSTc [77]. In the same study infections with JCPyV and JC pseudovirus (PsV) of JC type 1 (Mad-1 NCCR) and Mad-1 NCCR with type 3 VP1 sequence was unaffected by exogenous gangliosides [77]. These data implied gangliosides are not required for entry by genotypes 1 or 3 JCPyV. The Gorelik group showed that VLPs (type 3) containing the same sialic-acid binding pocket mutations discovered in PML patients were found to bind some gangliosides, and binding to target cells was unaffected by neuraminidase treatment [62]. Geoghegan and colleagues then demonstrated that VLPs of JCPyV genotype 2 and 3 wild-type (WT) or harboring a sialic acid binding mutation (S269F or L55F, respectively) can bind non-sialylated glycosaminoglycans (GAGs) on SFT cells (gliosarcoma). They also showed pseudoviruses (type 2 and 3, WT and sialic acid mutant) use GAGs for transduction in ART (ovarian tumor), SFT, and 293TT (transformed kidney) cells [84]. They hypothesized that VP1 receptor binding switches after the major capsid protein mutates from WT to sialic acid binding deficiency. While interesting, this has not yet been confirmed with crystallographic studies, live virus, recapitulated in the genotype 1 background, or completed with relevant permissive cell lines such as the commonly used SVG-A (transformed glial cells) or primary astrocytes. There is also some evidence that adipocyte plasma membrane-associated protein (APMAP) facilitates JCPyV (genotype 1) infection, though it is unclear whether it facilitates attachment or entry [85]. APMAP is an *N*-linked glycosylated type I transmembrane protein found in a variety of tissue types and could be an interesting avenue of research regarding JCPyV receptor-mediated infection in the brain [86,87].

During the second step of infectious entry JCPyV interacts with the 5-hydroxytryptamine type 2 receptor (5-HT_2_R) family that consists of three isoforms—2A, 2B, and 2C [88,89,90]. This interaction induces clathrin-dependent endocytosis by a β-arrestin mediated signaling pathway [90,91,92,93]. Once internalized the virus undergoes a series of trafficking and uncoating events before arriving at the nuclear compartment for transcription, genome replication, and assembly [94]. Table 1 summarizes research studies regarding JCPyV genotypes, receptors, and relevant publications.

Overall, diversity in VP1 sequences between viral genotypes and dissimilar receptor distribution on cell types studied could explain differential virus attachment factors but more direct comparison research is needed to clarify differences, define interactions for WT and sialic acid binding deficient viruses, and confirm requirements for productive infection. Currently, it is clear and well-established JCPyV (WT) requires LSTc and 5-HT_2_Rs for productive, receptor-dependent infection. However, this defined mechanism does not explain how JCPyV bypasses CNS barriers, whether sialic-acid binding deficient mutant viruses are infectious, or how JCPyV infects receptor-null cells like oligodendrocytes and astrocytes.

## 6. Extracellular Vesicles

Extracellular vesicles (EVs) are small, bilipid membrane-bound vesicles released from cells [96]. EV is a broad term for all vesicles released including exosomes, microvesicles, secretory autophagosomes, and apoptotic bodies [96,97,98]. EV sizes range from approximately 50 to 1000 nm and often have overlapping protein, lipid, and glycan profiles making it difficult to separate and characterize specific EVs [96,98]. EVs were initially characterized as trash released from the cell membranes until the mid-late 80 s when a group discovered that transferrin was released in EVs from reticulocytes during maturation and termed these exosomes [99,100]. EVs contain a myriad of proteins, lipids, and genetic material and research has boomed since the discovery that cargo can be selectively packaged into vesicles and functional in a target cell [96,97,98,101,102,103]. Interestingly, several non-enveloped viruses have been shown to exploit EVs to aid in immune evasion, increase tissue tropism, and facilitate en bloc infections [14,15,16,104,105,106,107,108,109,110]. There is also evidence enveloped viruses use EV pathways to disseminate viral proteins to neighboring cells [110,111,112,113,114,115,116,117,118,119,120,121]. This novel propagation of viral proteins and complete, infectious virus creates an obstacle to anti-viral therapeutics and treatments.

## 7. JC Polyomavirus Receptor-Independent Infection

Recent work from our lab examined the role of extracellular vesicles (EVs) in JCPyV (type 1) infection [15,16]. JCPyV was found enclosed within EVs and attached to the exterior. These virus positive EVs are infectious, resistant to anti-JCPyV antisera, and can infect target cells in a virus receptor-independent manner [15,16]. Importantly, JC pseudoviruses (type 1) containing one of the more common sialic-acid binding deficient mutations discovered in PML patients (L54F or S268F) were incapable of transducing naïve cells whereas the EV-associated PsV could [15]. This implies these mutant viral particles detected in patients may still spread by extracellular vesicles and contribute to disease. This work suggests EVs could cloak JCPyV from immune recognition and increase cellular tropism to receptor-lacking cells like oligodendrocytes and astrocytes.

## 8. JC Polyomavirus(+) Extracellular Vesicle Dissemination to the Brain Parenchyma

Overcoming either the blood-brain barrier (BBB) or the blood-cerebral spinal fluid barrier (BCSFB) to infect the brain parenchyma is important to understanding JCPyV disease progression. One hypothesis centers around bone marrow-derived cells. There is evidence JCPyV can persist in bone marrow and infect bone marrow-derived cells such as B cells [52,53,122]. B cells contain reassortment machinery and may provide means for JCPyV genome rearrangement and transport from sites of persistence to the central nervous system [38,52]. Immune cells are also known to monitor and interact with the central nervous system at the CNS barriers like the choroid plexus, BBB, and dura mater [123]. Other potential sites of viral persistence can include tonsils [50,53]. This site greatly reduces potential travel distance for JCPyV neuroinvasion. Underlying disease conditions (i.e., uncontrolled HIV or MS) also disrupt the BBB and might easily allow JCPyV infection of brain parenchyma by a hematogenous route [38,51].

**Figure 3:**
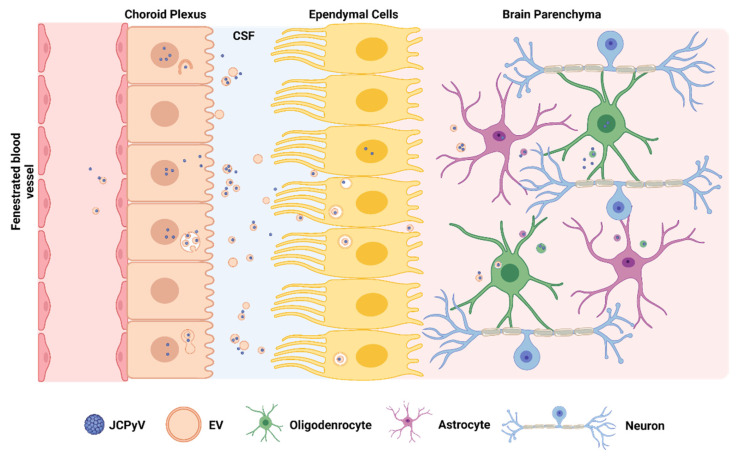
Hypothetical neuroinvasion of JCPyV via the blood-cerebrospinal fluid barrier. JCPyV and/or JCPyV(+) EVs can travel from a blood vessel to choroid plexus epithelial cells (CPE). CPEs are productively infected by JCPyV and package virus into EVs for dissemination to the CSF. JCPYV(+) EVs can travel to/through the ependymal layer (undefined) and invade the brain parenchyma to infect astrocytes and oligodendrocytes. Schematic created with BioRender.com (accessed on 18 April 2022).

Our lab presented another possibility via the BCSFB and extracellular vesicles [16,18,39]. The choroid plexus composes the BCSFB and is the major mediator of communication between the blood and cerebral spinal fluid [124]. We identified that primary choroid plexus epithelial cells are permissive to JCPyV in vitro and can produce JCPyV(+) EVs that are efficiently internalized by SVG-A cells via clathrin-dependent endocytosis or macropinocytosis [16,125]. We hypothesize the proximal position of the choroid plexus to ependymal cells and the brain parenchyma gives it optimal potential for JCPyV(+) EV dissemination into the brain (depicted in Figure 3) [124,126]. In fact, though exact mechanisms are still unclear EVs have already been demonstrated to cross the blood-CNS barriers into brain parenchyma [126,127,128]. In support of this idea the choroid plexus was recently shown to harbor JCPyV in patients [129]. JCPyV has also been detected associated with EVs purified from PML patient plasma, serum, and CSF [130]. This presents another possible, non-mutually exclusive mode of viral dissemination in the CNS. Understanding how virus associated EVs are created may provide potential target(s) for PML prevention.

## 9. Biogenesis of Extracellular Vesicles

Each type of EV is derived from a specific pathway within the host cell and has an assortment of proteins critical to formation, trafficking, fusion, and release [96]. Exosomes are typically the smallest EVs, derived from endosomes that undergo intralumenal vesicular budding to create multivesicular bodies (MVBs) [131,132]. MVBs are targeted for degradation or fusion with the plasma membrane, releasing the internal vesicles to the external space (now termed exosomes) [133]. Exosome production relies on several, non-mutually exclusive production pathways. A well-known and often-studied pathway involves sphingomyelinases [134,135,136]. Neutral sphingomyelinase 2 (nSMase2) acts at endosomal membranes to cleave sphingomyelin to ceramide and phospholipids [134]. Ceramide molecules packed together in a membranecan induce negative membrane curvature [137]. Exosomes can also rely on tetraspanins that bind and interact with one another and other proteins [138,139]. These interactions form tetraspanin-enriched microdomains implicated in negative membrane curvature and cargo loading [138,139,140,141,142]. Another exosome biogenesis pathway includes endosomal sorting complexes required for transport (ESCRT) proteins [143,144,145,146,147]. This series of five protein complexes is recruited sequentially to sort and load cargo, induce membrane curvature, force membrane pinching, and release a vesicle [146,147]. ESCRT proteins are also implicated in microvesicle budding [148,149].

**Figure 4:**
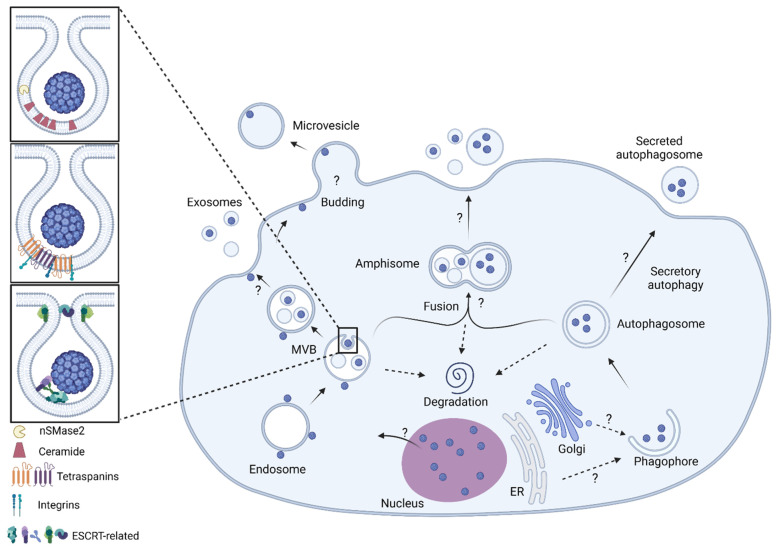
Potential JCPyV(+) EV biogenesis pathways. Exosomes, microvesicles, and secretory autophagosomes and their general biogenesis pathways are shown. Exosomes are produced by three non-mutually exclusive methods using (1) nSMase2 (top panel), (2) tetraspanins (middle panel), or (3) ESCRT-related proteins (bottom panel) to induce negative membrane curvature and produce multivesicular bodies (MVBs). MVBs can be directed for fusion with the plasma membrane by proteins (including RAB27A), for degradation, or for fusion with an autophagosome, producing an amphisome (middle). Amphisomes may be targeted for secretion by RAB27A [158,159,160]. Autophagosomes can be directed for secretion by the actions of RAB8A or GRASP65 (mechanism not well-defined) or targeted for degradation [155,156,157,158]. Nuclear escape for JCPyV is unknown. Schematic created with BioRender.com (accessed on 18 April 2022).

Autophagosomes are formed by a complex network of proteins that induce phagophore formation and maturation. The unconventional secretion pathway targets autophagosomes for the plasma membrane [150,151,152,153]. Proteins specific to secretory autophagosomes are crucial in targeting and mediating fusion with the plasma membrane [154,155,156,157,158,159,160]. Autophagosomes can also merge with MVBs to create an amphisome that is either targeted for degradation or fusion with the plasma membrane to release the internal contents [152,153,158,159,160,161]. Interestingly, many of these pathways and proteins are exploited by enveloped and nonenveloped viruses alike. Figure 4 depicts some potential virus-EV biogenesis methods.

## 10. Virus-EV Biogenesis Pathways

Enveloped viruses regularly use an assortment of EV related proteins to help in packaging, budding, and targeting during their life cycles [110]. For instance, several ESCRT related proteins are implicated in HIV budding and cytomegalovirus maturation [162,163,164], tetraspanins play a role in HIV, herpes simplex virus-1, and influenza virus infections [113,165,166], and β-coronaviruses like SARS-CoV-2 were recently shown to use secretory autophagy pathways for cellular escape [115]. Importantly, there are instances of viral proteins, mRNA, and microRNAs disseminated in EVs to uninfected cells [110,116,118,119,120,167,168,169,170,171]. Many research groups are pushing to understand which EV biogenesis pathways are exploited by viruses and how viral cargo is packaged into EVs.

**Figure 5:**
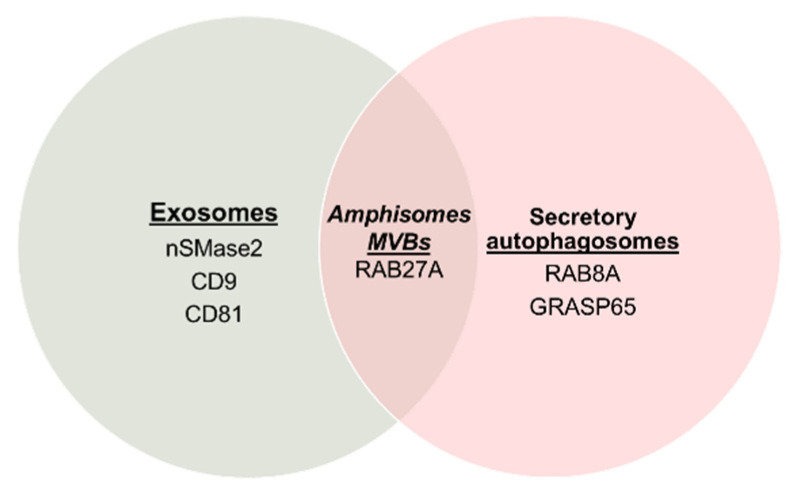
Venn diagram of EVs and associated proteins important in producing JCPyV(+) EVs. Exosomes dependent on nSMase2, CD9, and/or CD81 contribute to JCPyV(+) EV population. Secretory autophagosomes directed by RAB8A and/or GRASP65 produce JCPyV(+) EVs. RAB27A is important in JCPyV(+) EV biogenesis, either in targeting amphisomes and/or MVBs.

Our lab identified that SVG-A released JCPyV(+) EVs are heterogeneous populations including exosomes and secretory autophagosomes. Using chemical and genetic methods, we demonstrated that JCPyV(+) EVs were dependent on nSMase2, tetraspanins CD9 and CD81, small GTPases RAB27A and RAB8A, and the Golgi restacking protein 65 (GRASP65, also known as GoRASP1). Chemical inhibition or genetic depletion of nSMase2 reduced JCPyV spread. Knockdown or knockout nSMase2 cells decreased EV-mediated infection compared to control cells with no change to internalization by target cells. Knockdown of CD9 or CD81 similarly reduced viral spread, decreased EV-mediated infection, but internalization by target cells was unaffected. Knockdown of RAB8A, RAB27A, or GRASP65 reduced viral spread and decreased infectious EV production without differential uptake compared to controls, suggesting secreted autophagosomes contribute to the JCPyV(+) EV population [172]. Figure 5 outlines the proteins and their associated EV pathway.

Interestingly, seven ESCRT-related proteins were tested independently, none of which demonstrated an important role in EV-mediated infection. In fact, it seemed the full complement of ESCRT proteins did more to control JCPyV infection. Finally, it was noted that depletion of single proteins from any pathway did not alter virus-EV spatial relationships or size distribution of released EVs [172]. It is crucial to recognize that despite the current findings across the literature, many of these proteins may act across multiple EV biogenesis and secretion pathways. This implies there are multiple pathways JCPyV exploits for virus-EV biogenesis that may include complementary and/or compensatory mechanisms.

## 11. JCPyV and EV Purification and Characterization Methods

JC polyomavirus can spread as free virus, EV-associated, or cell-to-cell [15,16,39,173]. With virus receptor-dependent and -independent mechanisms possible, careful purification methods are required to ensure alignment between expectations and realities of viral entry research. JCPyV purification must be centered around complete removal of contaminating lipids, proteins, and DNA from the cell lysate. Typical methods include some combination of detergents, sonication, neuraminidase cleavage of sialic acid-attached glycolipids and/or glycoproteins, DNase digestion, lipid extraction(s), concentration through sucrose, and final separation through a gradient like iodixanol or cesium chloride [174,175,176]. It is important to note that concentrating through sucrose may remove some impurities, but extracellular vesicles and virus both pellet through a typical sucrose step [177,178]. Gradients can separate virus particles from EVs, but incomplete purification and separation may lead to misinterpreted results. Important characterization methods include electron microscopy, genome sequencing, and titer evaluation.

There are many EV purification methods and choosing the right method is challenging. Optimal EV methods sacrifice yield, purity, cost, and/or time depending on the sample [179]. Basic EV purification is based on either density or size. Common EV separation techniques include differential centrifugation, size exclusion chromatography, filtration, flow-field filtration, gradients, or a combination of methods [178,179,180,181,182]. Many labs use differential centrifugation to separate different sized EVs for ease of use, low cost, increased sample volume processing, and high yield, but sacrifice purity and time [180]. Samples are subjected to increasing centrifugal forces to clear debris and separate apoptotic bodies, large EVs, and small EVs [178]. After EV purification the sample must be characterized by several methods to confirm appropriate size distributions, concentration, morphology, and presence of EV markers and absence of contaminants [181,182]. Methods include nanoparticle tracking analysis, electron microscopy, and Western blot analysis [178,181,182]. Omics applications (e.g., proteomics, transcriptomics, metabolomics, and lipidomics) and functional studies help define content of EVs and functionality in other cells [181,182,183,184,185].

While JCPyV is typically purified from whole cell lysate and EVs from supernatant, there is still overlap of vesicle associated virus in lysate and free virus in the supernatant. With current technology, separation of virus and EVs can be cumbersome, imperfect, and result in low yield [186]. Overall, until new separation and purification methods are created and accessible, careful management and characterization of samples paired with clever functional studies help demonstrate validity of subsequent experimental results.

## 12. Discussion

Research has conclusively demonstrated JCPyV uses both virus receptor dependent and independent entry mechanisms. Receptor dependent entry requires the sialic acid containing attachment receptor LSTc followed by entry receptor(s) 5-HT_2_R. While recent work points to other potential receptors that may contribute to infection for different virus genotype backgrounds and/or cell types (i.e., types 1, 2, or 3 and gangliosides vs. APMAP), further work is needed to define these additional virus-receptor interactions and determine relevance to disease progression. Extracellular vesicles mediate a novel virus receptor-independent mechanism of JCPyV spread. EVs may be key to immune evasion, neuroinvasion, and infection of virus receptor-lacking oligodendrocytes and astrocytes. Further work examining nuclear escape or cargo loading mechanisms will be key to fully understanding JCPyV pathogenesis and may reveal druggable viral targets that would prevent EV association and decrease infectious JCPyV(+) EV production from host cells. It is also important to look at other virus models and understand how prevalent this potential viral dissemination mechanism might be. It was recently demonstrated that BK polyomavirus are found inside EVs and are internalized independently of sialic acid, suggesting BKPyV(+) EVs can also infect independently of viral receptors [14]. Interestingly, BKPyV EV-mediated infection was neutralized by patient-derived anti-BKPyV serum [14]. The work from our lab used rabbit-derived anti-JCPyV antisera, so it will be interesting to evaluate if JCPyV(+) EVs are neutralized by patient-derived serum and/or antibodies. Further research exploring potential associations between viruses and extracellular vesicles and the effect on virus propagation in vivo will be important to understanding the overall impact of EVs.

Importantly, the recognition that JCPyV (and other viruses) may undergo virus receptor-dependent and -independent infection points to a need for meticulous purification methods. Virus-specific purification methods are vital to understanding and appropriately interpreting virus-host interactions such as receptor binding, infectious entry, and early trafficking. At the same time, we must still consider the EV associated viral population that may play important roles in vivo [90,187]. JCPyV can exist in both nonenveloped and quasi-enveloped forms within a host and ignoring either population of JCPyV reduces the chances of discovering effective therapeutics for preventing and treating JCPyV-induced disease [130,188]. Overall, the association of nonenveloped viruses and extracellular vesicles blurs the line between enveloped vs. nonenveloped classification. Defining the interplay between JCPyV and EVs is central to appreciating viral pathogenesis, disease progression, and development of therapeutics.

## Figures and Tables

**Table 1:** Summary of virus-receptor studies for rearranged JCPyV types. Abbreviations are as follows: VLP, Virus-Like Particles; PsV, Pseudovirus; LSTc, LactoSeries Tetrasaccharide C; GAG, GlycosAminoGlycans; 5-HT_2_, 5-HydroxyTryptamine 2; APMAP, Adipocyte Plasma Membrane-Associated Protein. Type is synonymous with genotype.

Receptor	Pentamer	VLP	PsV	Virus
**LSTc**	Type 1 [76], 3 [77]	---	Type 1, 3 [77]	Type 1 [76,77], 3 [77]
**Gangliosides**	Type 1, 3 [77]	Type 1 [81], 2 & 3 [84], 3 [62]	Type 1 & 3 [77], 2 & 3 [62,84]	Type 1, 3 [77]
**GAGs**	---	Type 2, 3 [84]	Type 2, 3 [84]	---
**5HT_2_A/B/C**	---	---	Type 1 [90]	Type 1 [88,89,90,95]
**APMAP**	---	---	---	Type 1 [85]

## Data Availability

Not applicable.
